# Identification of BST2 Contributing to the Development of Glioblastoma Based on Bioinformatics Analysis

**DOI:** 10.3389/fgene.2022.890174

**Published:** 2022-07-05

**Authors:** Yang Kong, Zhiwei Xue, Haiying Wang, Guangqiang Cui, Anjing Chen, Jie Liu, Jian Wang, Xingang Li, Bin Huang

**Affiliations:** ^1^ Department of Neurosurgery, Qilu Hospital, Cheeloo College of Medicine and Institute of Brain and Brain-Inspired Science, Shandong University, Jinan, China; ^2^ Jinan Microecological Biomedicine Shandong Laboratory and Shandong Key Laboratory of Brain Function Remodeling, Jinan, China; ^3^ Neurological Care Unit, The Affiliated Yantai Yuhuangding Hospital of Qingdao University, Yantai, China; ^4^ Department of Neurosurgery, The Affiliated Yantai Yuhuangding Hospital of Qingdao University, Yantai, China; ^5^ Department of Anesthesiology, The Affiliated Yantai Yuhuangding Hospital of Qingdao University, Yantai, China; ^6^ Department of Biomedicine, University of Bergen, Bergen, Norway

**Keywords:** glioblastoma, bioinformatics, BST2, interferon, immune

## Abstract

Rigorous molecular analysis of the immune cell environment and immune response of human tumors has led to immune checkpoint inhibitors as one of the most promising strategies for the treatment of human cancer. However, in human glioblastoma multiforme (GBM) which develops in part by attracting immune cell types intrinsic to the human brain (microglia), standard immunotherapy has yielded inconsistent results in experimental models and patients. Here, we analyzed publicly available expression datasets to identify molecules possibly associated with immune response originating from or influencing the tumor microenvironment in primary tumor samples. Using three glioma datasets (GSE16011, Rembrandt-glioma and TCGA-glioma), we first analyzed the data to distinguish between GBMs of high and low tumor cell purity, a reflection of the cellular composition of the tumor microenvironment, and second, to identify differentially expressed genes (DEGs) between these two groups using GSEA and other analyses. Tumor purity was negatively correlated with patient prognosis. The interferon gamma-related gene *BST2* emerged as a DEG that was highly expressed in GBM and negatively correlated with tumor purity. *BST2* ^*high*^ tumors also tended to harbor *PTEN* mutations (31 vs. 9%, *BST2* ^*high*^ versus *BST2* ^*low*^) while *BST2* ^*low*^ tumors more often had sustained *TP53* mutations (8 versus 36%, *BST2* ^*high*^ versus *BST2* ^*low*^). Prognosis of patients with *BST2* ^*high*^ tumors was also poor relative to patients with *BST2* ^*low*^ tumors. Further molecular in silico analysis demonstrated that high expression of *BST2* was negatively correlated with CD8^+^ T cells but positively correlated with macrophages with an M2 phenotype. Further functional analysis demonstrated that BST2 was associated with multiple immune checkpoints and cytokines, and may promote tumorigenesis and progression through interferon gamma, IL6/JAK/STAT3 signaling, IL2/STAT5 signaling and the TNF-α signaling *via* NF-kB pathway. Finally, a series of experiments confirmed that the expression of BST2 can be significantly increased by IFN induction, and knockdown of BST2 can significantly inhibit the growth and invasion of GBM cells, and may affect the phenotype of tumor-associated macrophages. In conclusion, BST2 may promote the progression of GBM and may be a target for treatment.

## Introduction

Glioblastoma multiforme (GBM) is the most common and lethal tumor type in the central nervous system (CNS), and exhibits many aggressive tumor characteristics such as rapid growth, invasion, and genetic heterogeneity (Appin and Brat, 2015; Reifenberger et al., 2017). Moreover, lesions cannot be completely removed leading to the inevitability of recurrence, and the presence of the blood-brain barrier complicates access of various molecular therapies to the tumor bed (Aldape et al., 2019). Thus, despite the multimodal therapy of surgery, radiation and chemotherapy, most patients with GBM survive less than 2 years following primary diagnosis (Stupp et al., 2005).

One of the more promising treatment strategies for aggressive human cancers has been immunotherapy (Medrano et al., 2017; Singh and McGuirk, 2020). Current approaches include cellular immunotherapy, such as chimeric antigen receptor T cells (CAR-T therapy), and strategies that trigger the immune response, such as antibodies, cytokines, immune checkpoint inhibitors, and oncolytic viruses (Togashi et al., 2019; Krishna et al., 2020; Fares et al., 2021). Targeting mutant *TP53* has also been an attractive approach to modulate immune response in cancer immunotherapy, and *TP53* and genes in its pathway are frequently mutated during the development of GBM (Shiraishi et al., 2002; Chasov et al., 2021). While these therapies have been successful in some primary tumor types such as melanoma, lung and colon cancers, treatment of GBM patients with immunotherapies has been less effective, in part due to a unique and complex tumor microenvironment which consists of a variety of stromal cell types, including fibroblasts, adipocytes, endothelial cells and immune cells of either intrinsic (microglia) or peripheral origin, and the extracellular matrix (Buerki et al., 2018; Shergalis et al., 2018). Many of these cell types promote tumor development, such as tumor-associated macrophages (Pyonteck et al., 2013) and tumor-associated fibroblasts which, for example, secrete the protumorigenic chemokine CXCL14 (Zhang et al., 2019a).

Molecular characterization of GBM samples with an emphasis on tumor microenvironment might lead to the identification of different molecules that could be targeted for therapy, particularly with respect to the infiltrating immune cells types in human gliomas (Reifenberger et al., 2017). Here, we analyzed expression data in publicly available databases to distinguish between profiles of tumors with a high versus low stromal cell content, or low purity versus high purity tumors (Zhang et al., 2017). Analysis to identify differentially expressed genes (DEGs) between these two groups of tumors yielded *BST2*, a gene associated with immune cell types/function (Swiecki et al., 2012). Our analysis provides a path towards mining publicly available databases with the goal of targeting molecules unique to the GBM tumor microenvironment and may improve the efficacy of anti-GBM immunotherapy.

## Materials and Methods

### Data Source

Analysis was performed on expression data obtained from three publicly available glioma datasets, GSE16011, Rembrandt-glioma and TCGA-glioma. Expression data collected on the Affymetrix GeneChip Human Genome U133 Plus 2.0 Array was obtained from GSE16011 (*n* = 276 cases WHO grades II-IV, *n* = 8 non-neoplastic brain tissue samples; https://www.ncbi.nlm.nih.gov/geo/query/acc.cgi?acc=GSE16011) (Gravendeel et al., 2009) and Rembrandt-glioma datasets (*n* = 474 gliomas; https://caintegrator.nci.nih.gov/rembrandt/) (Gusev et al., 2018). For the TCGA-glioma dataset (*n* = 699 cases; https://gdc.cancer.gov/) (Cancer Genome Atlas Research Network, 2008), normalized expression data obtained through RNA sequencing and the mutation data generated through DNA sequencing were used in our analysis. Clinical data for all patients were obtained from the corresponding data portal.

### Cell Lines and Cultures

Normal human astrocytes (NHA) and GBM cell lines U251, LN229, A172 and human monocyte leukemia cell line (THP-1) were provided by the Chinese Academy of Sciences Cell Bank (Shanghai, China) and authenticated by short tandem repeat (STR) profiling. The *Mycoplasma* PCR Detection Kit was used to detect *Mycoplasma* contamination. The cells are anchorage-dependent cells in the absence of any stress. Cells were cultured in complete medium: Dulbecco’s modified Eagle’s medium (DMEM, Thermo Fisher Scientific; Waltham, MA, United States), 10% fetal bovine serum (FBS, Thermo Fisher Scientific), streptomycin (100 U/ml) and penicillin (100 U/ml). THP-1 cells were treated with 200 nM phorbol-12-myristate-13-acetate (PMA, Sigma-Aldrich; St. Louis, MO, United States) for 24 h to allow for the differentiation into macrophages in 6-well plates. The cells were cultured in a humidified incubator (HERAcell 240i, Thermo Fisher Scientific) at 37°C and 5% CO_2_.

### Preparation of Experimental Reagents

Interfering RNA sequences (siRNA) were synthesized (GenePharma, Shanghai, China). Before use, the siRNA powder was centrifuged, dissolved in DEPC water, aliquoted and stored at −20°C. Recombinant human IFN-γ protein was provided by T&L Biological Technology (Beijing, China). IFN-γ protein was dissolved in sterile PBS, aliquoted and stored at −20°C.

### Bioinformatics Analysis

The Estimation of STromal and Immune cells in Malignant Tumor tissues using Expression data (ESTIMATE) R package was used to calculate tumor purity and the presence of infiltrating stromal/immune cells in tumor tissues based on transcriptomic data (Yoshihara et al., 2013). TIMER (https://cistrome.shinyapps.io/timer/) was used for systematic analysis of immune infiltrates (Li et al., 2020). The CIBERSORT database (https://cibersort.stanford.edu/runcibersort.php) was used to estimate the fraction of 22 immune cell types in a mixed cell population (Newman et al., 2015). Gene Set Enrichment Analysis (GSEA) was performed with the JAVA program (http://www.broadinstitute.org/gsea, version 4.0.3), using | NES |> 1, p-val < 0.05 as the criterion for the results (Subramanian et al., 2005). MSigDB gene set collections were used in the analysis of the expression profile data of different groups to correlate the enriched corresponding genes obtained from different molecular signatures to biological processes. R “edgeR” package was used to identify DEGs based on a threshold false discovery rate (FDR) of <0.05 and an absolute log2 fold change (FC) of >1 (Robinson et al., 2010).

The variation data and corresponding clinical data from the TCGA-glioma dataset were analyzed with the R “maftools” package, and the top 20 genes with the highest frequency of mutations in different groups were displayed in a waterfall chart (Mayakonda et al., 2018). Kaplan–Meier survival curves, expression difference box plots and histograms were generated using GraphPad Prism version eight software (San Diego, CA, United States). Intersecting genes were identified using Database for Annotation, Visualization and Integrated Discovery (DAVID, https://david.ncifcrf.gov/) (Huang et al., 2009) for Kyoto Encyclopedia of Genes and Genomes (KEGG, https://www.genome.jp/kegg/) pathway enrichment analysis (Ogata et al., 1999). KEGG pathway enrichment bubble charts, gene expression heat maps, correlation coefficient maps and chord diagrams were created with ggplot2 (Wickham, 2009), Pheatmap (Raivo, 2012), Corrplot (Wei et al., 2017) and Circlize package (Gu et al., 2014) in R Version 3.5.1, respectively.

### RNA Interference

SiRNAs targeting BST2 were transfected into cells with Lipofectamine 2000 reagent (Thermo Fisher Scientific) according to the protocol. After 4 h, fluorescently labeled RNA was used to detect transfection efficiency. Knockdown efficiency was evaluated 48 h after transfection by qRT-PCR or immunoblotting. SiRNA sequences used are the following:

si-Control: 5′-UUC​UCC​GAA​CGU​GUC​ACG​UTT-3′;

si-BST2-1: 5′-GCA​UCU​ACU​UCG​UAU​GAC​UTT-3′;

si-BST2-2: 5′-GGA​UAG​GAA​UUC​UGG​UGC​UTT-3′;

si-BST2-3: 5′-GAG​CGA​CUG​AGA​AGA​GAA​ATT-3′.

### Production of Conditioned Supernatant

U251 cells were seeded 5 × 10^6^ cells/well in 6-well plates and allowed to adhere overnight. They were then extensively washed and incubated for 48 h with fresh complete DMEM containing si-Control or si-BST2. Supernatants were collected, centrifuged at 5000 g, filtered using a 0.2 μm filter (Millipore) and stored at −80°C until use.

### Real-Time PCR

Total RNA was prepared from treated cells using TRIzol (Thermo Fisher Scientific). Briefly, after centrifugation, the aqueous layer was transferred to a fresh eppendorf tube, and isopropanol was added to precipitate total RNA. cDNA was generated from total RNA (1 µg) using the ReverTra Ace qPCR RT Kit (TOYOBO, Osaka, Japan). qRT-PCR was performed with SYBR Green Master (Roche, Basel, Switzerland) on the CFX96 Real Time PCR Detection System (Roche). *ACTB* mRNA was used to normalize mRNA expression. The results are representative of at least three independent experiments. The sequences of the PCR primers used are the following: IL10-Forward: 5′-CCA​GAC​ATC​AAG​GCG​CAT​GT-3′, Reverse: 5′-GAT​GCC​TTT​CTC​TTG​GAG​CTT​ATT-3′; TGF-β- Forward: 5′-GCA​ACA​ATT​CCT​GGC​GAT​ACC-3′, Reverse: 5′-ATT​TCC​CCT​CCA​CGG​CTC​AA-3′; TNF-α- Forward: 5′- GAG​GCC​AAG​CCC​TGG​TAT​G-3′, Reverse: 5′-CGG​GCC​GAT​TGA​TCT​CAG​C-3′; BST2- Forward: 5′-ATG​GAA​GAC​GGG​GAT​AAG​CG-3′, Reverse: 5′-GGA​GAT​GGG​TGA​CAT​TGC​GA-3′; ACTB- Forward: 5′-CAT​GTA​CGT​TGC​TAT​CCA​GGC-3′, Reverse: 5′-CTC​CTT​AAT​GTC​ACG​CAC​GAT-3′.

### Western Blotting

Harvested cells were lysed with heat denaturation in RIPA cell lysis buffer supplemented with phenylmethanesulfonyl fluoride (PMSF, Beyotime, Shanghai, China). Protein lysates (20 μg) were run on SDS-PAGE, and proteins were transferred to polyvinylidene difluoride (PVDF) membrane (0.22 μm, Millipore; Danvers, MA, United States). Blots were incubated with primary antibodies against BST2 (Proteintech, Wuhan, China) and ACTB (Cell Signaling Technology; Danvers, MA, United States). HRP-labeled goat anti-rabbit secondary antibodies were purchased from Zhongshan Golden Bridge Bio-technology (Beijing, China). Specific proteins were detected with enhanced chemiluminescence (ECL, Millipore). Luminous intensity was detected with the Chemiluminescence Imager (Bio-Rad ChemiDoc XRS+, Bio-Rad; Hercules, CA, United States) according to the manufacturer’s protocol. Band density was measured (ImageJ software) and normalized to ACTB.

### Cell Viability Assay

Cell viability was assessed using the Cell Counting Kit-8 assay (CCK-8, Dojindo; Kumamoto, Japan). GBM cells (4 × 10^3^) were seeded into the wells of 96-well plates (Corning, Corning, NY, United States) and cultured in a humidified incubator at 37°C and 5% CO_2_. After 12 h, the medium was replaced with 100 μl of fresh culture medium containing corresponding treatment or control. At 24, 48, 72, and 96 h after dosing, GBM cells were incubated with 10 μl of CCK-8 reagent in 100 μl of serum-free DMEM at 37°C for an hour. The absorbance at 450 nm was measured using an EnSight Multimode Plate Reader (PerkinElmer, Singapore).

### 5-Ethynyl-2′-Deoxyuridine Cell Proliferation Assay

GBM cells (2 × 10^4^) were seeded into 24-well plates, allowed to attach, and treated. Following treatment, cell proliferation was measured using the EdU Apollo 567 Cell Tracking Kit (Ribo-bio, Guangzhou, China). Briefly, cells were incubated with EdU reagent (200 μM) for 2 h at room temperature, fixed with 4% paraformaldehyde for 20 min and permeabilized with 0.5% Triton X-100 for 10 min. Cells were rinsed with phosphate buffered saline (PBS) three times, incubated with 100 μl of Apollo reagent for 30 min and stained with 4′,6-diamidino-2-phenylindole (DAPI). Three fields of view were randomly selected for counting EdU-positive cells, and three independent experiments were performed.

### Cell Invasion Assay

Transwell assays were performed in matrigel-coated (BD Biosciences, San Jose, CA, United States) Transwell chambers (8 μm pores, Corning). Cells (2 × 10^4^) in DMEM medium (200 μl) were seeded in the top chamber. The lower chamber was filled with medium (600 μl) containing 30% FBS. Chambers were incubated for 24 h. Cells that migrated to the lower surface were fixed with 4% paraformaldehyde (Solarbio, Beijing, China), stained with crystal violet (Solarbio) for 15 min and counted under bright field microscopy. Images were acquired from five random fields in each well.

### Statistical Analysis

Each assay was performed at least three times independently. Data were reported as the mean ± SD. Quantitative data were first tested for normal distribution. If the data met the criteria for normal distribution, differences were assessed between two groups using a Student’s t test and between multiple groups using one-way ANOVA. Otherwise, non-parametric tests were chosen to detect the differences. The association between glioma purity and continuous variables was assessed using Spearman rank correlation. The log-rank test was used to assess the statistical significance between groups. Patients with missing information were excluded from corresponding analysis. All statistical analyses were conducted using R Version 3.5.1 and GraphPad Prism version eight software (San Diego, CA, United States). Differences were considered to be significant at the following *p*-values: *p < 0.05; **p < 0.01; ***p < 0.001.

## Results

### High Tumor Purity is Associated With Better Prognosis in Human Glioma

**FIGURE 1:**
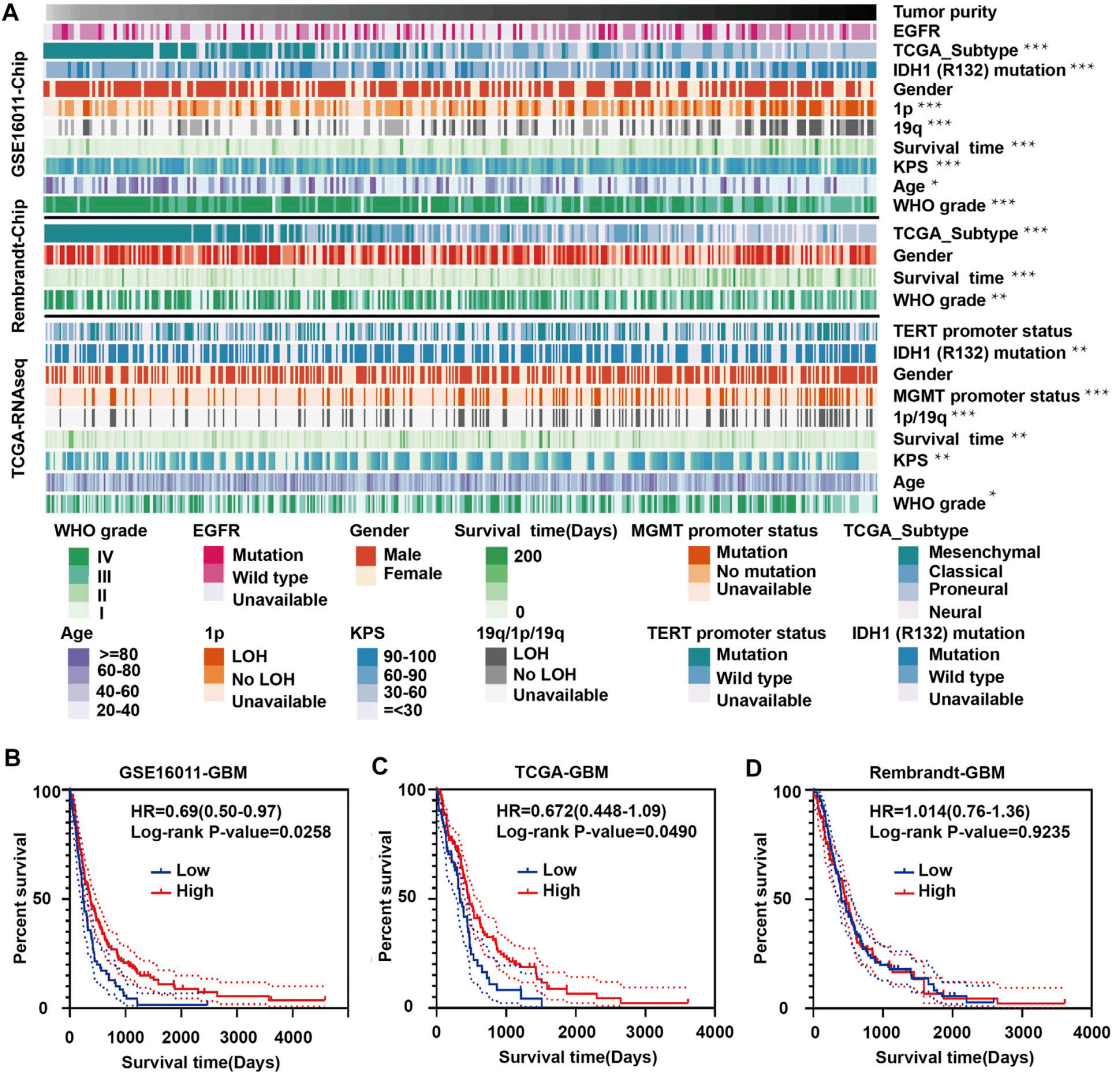
Landscape of clinical and molecular characteristics associated with glioma purity. **(A)** Diagram showing clinical and molecular data for a total of 1449 samples from all three datasets (GSE16011, Rembrandt and TCGA-glioma) arranged on the basis of increasing tumor purity. The status of clinical and molecular characteristics is designated based on shades of colors. The relationship between glioma purity and patient characteristics was evaluated under the following conditions: (a) the distribution of glioma purity was assessed using the Student’s t test between two groups; (b) the association between glioma purity and continuous variables was assessed using Spearman correlation tests; and (c) the distribution of glioma purity between several groups was assessed using one-way ANOVA. Kaplan–Meier survival curves for GBM patients with high and low purity tumors in **(B)** GSE16011, **(C)** TCGA-glioma and **(D)** Rembrandt. The statistical differences in the figure are highlighted as follows: ^∗^ *p* < 0.05; ^∗∗^ *p* < 0.01; ^∗∗∗^ *p* < 0.001.

To explore the relationship between tumor purity and clinical characteristics, we ranked the tumor purity of the samples in the GSE16011, Rembrandt and TCGA-glioma datasets from low to high along with clinical characteristics, including WHO grade, EGFR, IDH1, and 1p/19q status, survival time, and age (Figure 1A). The correlation between tumor purity and clinical characteristics was calculated using a previously described method (Zhang et al., 2017). Higher WHO grade was associated with lower tumor purity, while longer overall survival and higher KPS were associated with higher tumor purity. Tumor purity based on the four molecular glioma subtypes was often lower in mesenchymal tumors relative to proneural and classical tumors. In contrast, tumor purity was higher in tumors with 1p/19q codeletion, *IDH1* mutation (R132) and/or *MGMT* promoter mutation.

Some results were inconsistent between data sets. For example, we found a negative correlation between glioma purity and age at diagnosis in the GSE16011 dataset, but no such correlation was observed in the other two datasets. These results corroborate a study by Chuanbao Zhang et al. (2017).

We further examined the significance of high- and low-purity tumors using Kaplan-Meier analysis to determine the overall survival of patients in the three data sets. Longer overall survival was associated with high-purity tumors based on the GSE16011 and TCGA datasets (Figures 1B,C). However, we found no significant difference between the two groups using the Rembrandt dataset (Figure 1D). These results may be due to the small sample size and sample bias.

### The Interferon Gamma Signature is Associated With Tumor Purity

**FIGURE 2:**
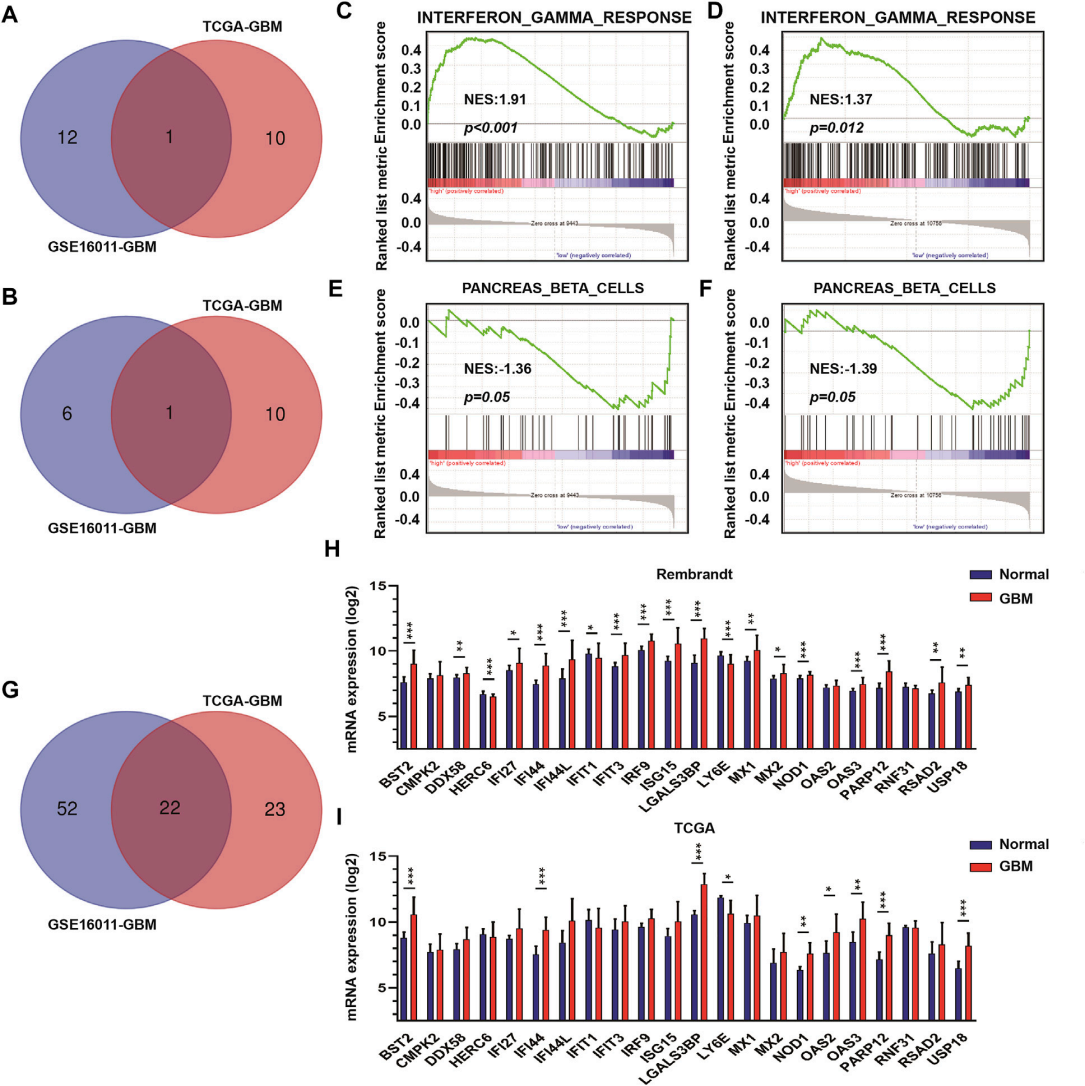
The interferon γ signature is associated with differences in GBM tumor purity. **(A)** Venn diagram displaying the intersection between up-regulated molecular signatures in GSE16011-GBM and TCGA-GBM datasets. **(B)** Venn diagram displaying the intersection between down-regulated molecular signatures in GSE16011-GBM and TCGA-GBM datasets. GSEA results for interferon γ response in **(C)** GSE16011-GBM and **(D)** TCGA-GBM datasets. GSEA results for pancreas beta cells in **(E)** GSE16011-GBM and **(F)** TCGA-GBM datasets. **(G)** Venn diagram displaying the intersection between core genes of the interferon gamma signature in GSE16011 and TCGA datasets. The expression levels of intersecting genes in normal brain and GBM tissue samples in **(H)** Rembrandt and **(I)** TCGA datasets. The statistical differences in the figure are highlighted as follows: ^∗^ *p* < 0.05; ^∗∗^ *p* < 0.01; ^∗∗∗^ *p* < 0.001.

To identify DEGs between GBM high-purity and low-purity tumors, we first used the “edgeR” package to analyze the molecular profiles of GBMs in GSE16011 and TCGA datasets (FDR <0.05, | log2fold change |> 1). No DEGs were obtained. When we switched to the more sensitive, genome-wide GSEA method, we found that 13 molecular signatures were up-regulated in the high-purity tumor group while seven were down-regulated in the low-purity group using the GSE16011 dataset. In the TCGA dataset, we found 11 up-regulated and down-regulated molecular signatures associated with high and low purity tumor groups, respectively. The intersection of the up-regulated and down-regulated molecular signatures of the two datasets yielded profiles associated with interferon gamma response and pancreas beta cells (Figures 2A,B respectively) as well as in GSEA (interferon gamma response, Figures 2C,D, and pancreas beta cells, Figures 2E,F in GSE16011 and TCGA data sets, respectively).

Considering that interferon gamma, as the only type II interferon, has significant antiviral and antitumor properties and shows fewer side effects (Ni et al., 2013; Li et al., 2018; Song et al., 2019), we focused on the relationship between genes in the interferon gamma molecular tags and tumor progression and purity. Analysis of the intersection of the core genes of the interferon gamma signature in the two datasets yielded several genes, which included *BST2*, *CMPK2*, *DDX58*, *HERC6*, *IFI27*, *IFI44*, *IFI44L*, *IFIT1*, *IFIT3*, *IRF9*, *ISG15*, *LGALS3BP*, *LY6E*, *MX1*, *MX2*, *NOD1*, *OAS2*, *OAS3*, *PARP12*, *RNF31*, *RSAD2*, and *USP18* (Figures 2G–I). We examined the expression of these genes in the Rembrandt and TCGA databases and selected those with consistent expression among the three datasets. Finally, we obtained a total of eight genes for further analysis, including *BST2*, *IFI44*, *LGALS3BP*, *LY6E*, *NOD1*, *USP18*, *OAS3*, and *PARP12*.

### High Expression of *BST2* is Associated With Worse Prognosis

**FIGURE 3:**
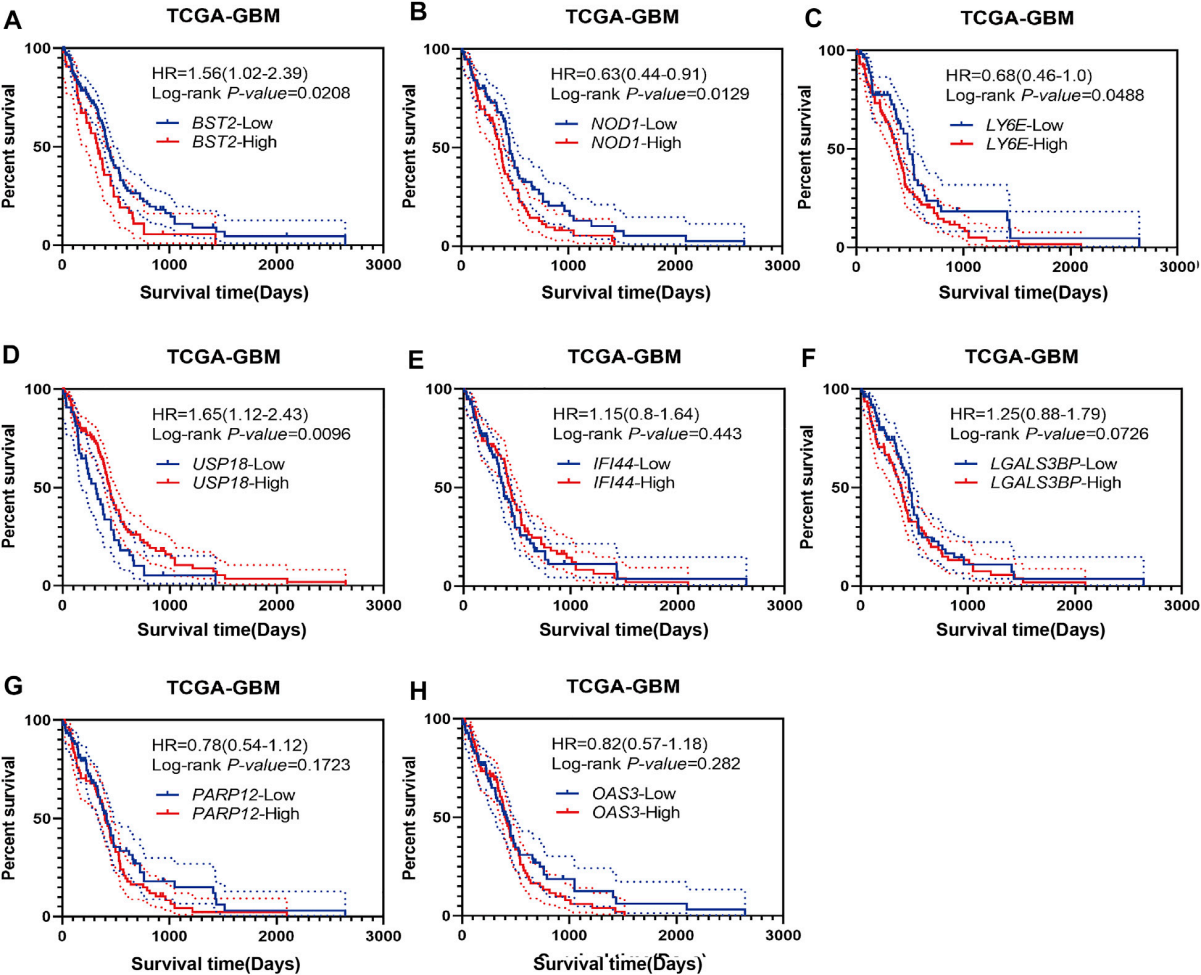
Prognostic analysis of intersecting genes in the TCGA-GBM dataset. Kaplan–Meier survival curves for patients based on high and low expression of intersecting genes in GBMs: **(A)** *BST2* ^*high*^ vs. *BST2* ^*low*^; **(B)** *NOD1* ^*high*^ vs. *NOD1* ^*low*^; **(C)** *LY6E* ^*high*^ vs. *LY6E* ^*low*^; **(D)** *USP18* ^*high*^ vs. *USP18* ^*low*^; **(E)** *IFI44* ^*high*^ vs. *IFI44* ^*low*^; **(F)** LGALS3BP^*high*^ vs. LGALS3BP^*low*^; **(G)** *PARP12* ^*high*^ vs. *PARP12* ^*low*^ and **(H)** *OAS3* ^*high*^ vs. *OAS3* ^*low*^. High and low expression is based on the median value of expression for that gene.

Using Kaplan-Meier analysis, we examined overall survival of patients in the TCGA-GBM dataset based on high and low expression of *BST2*, *IFI44*, *LGALS3BP*, *LY6E*, *NOD1*, *USP18*, *OAS3*, and *PARP12* in tumors. High and low expressions were determined based on the median expression values of the genes in the dataset. Groups showing high expression of *BST2*, *NOD1* or *LY6E* were associated with a poor prognosis (Figures 3A–C). In contrast, high expression of *USP18* was associated with a better prognosis (Figure 3D). There was no significant difference in overall survival of patients based on high and low expression of *IFI44*, *LGALS3BP*, *PARP12* or *OAS3* (Figures 3E–H). Due to the consistency of the results for *BST2* in multiple datasets and the ranking of core genes, we selected *BST2* for further analysis.

### Increased Expression of *BST2* is Associated With Characteristics of Higher Grade Gliomas

**FIGURE 4:**
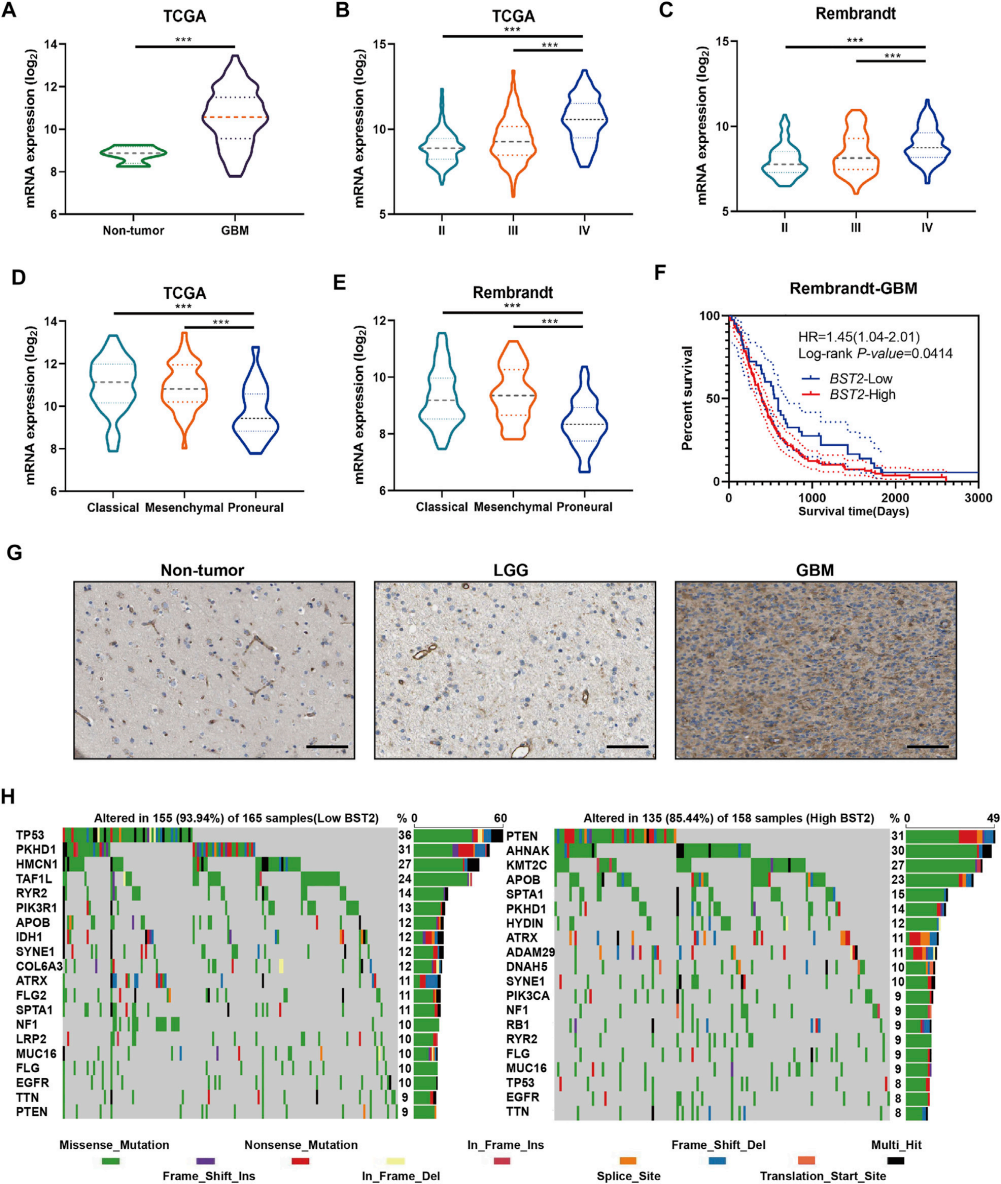
BST2 is associated with multiple clinical features. **(A)** *BST2* mRNA levels in non-neoplastic brain relative to GBM tissue samples in the TCGA-glioma dataset. *BST2* mRNA expression profiles based on pathological grade from the TCGA-Glioma dataset **(B)** and Rembrandt-Glioma datasets **(C)**. *BST2* mRNA expression profiles based on molecular subtype from the TCGA-Glioma dataset **(D)** and Rembrandt-Glioma datasets **(E)**. **(F)** Kaplan–Meier survival curves for GBM patients with *BST2* ^*high*^ and *BST2* ^*low*^ tumors using the Rembrandt database. **(G)** IHC of BST2 in non-neoplastic tissues, low grade glioma (LGG) and GBM. Scale bars: 100 µm. **(H)** Spectrum of somatic mutations in gliomas from *BST2* ^*low*^ and *BST2* ^*high*^ groups in the TCGA-glioma dataset. ^∗∗∗^ *p* < 0.001.

We next examined the expression of BST2 based on tumor grade and molecular characteristics of glioma histological and molecular subtypes. First, *BST2* in GBM samples was significantly increased compared with normal brain tissue (Figure 4A). Second, the expression level of *BST2* significantly increased with increasing pathological grade in tumors from TCGA and Rembrandt databases (Figures 4B,C). Third, *BST2* expression was highest in GBMs with the classical molecular subtype, followed by the mesenchymal subtype, and lowest in the proneural subtype based on the data in TCGA and Rembrandt databases (Figures 4D,E). Fourth, patients from the Rembrandt database with high *BST2* expressing tumors also exhibited worse prognosis (Figure 4F). Finally, immunohistochemical results from The Human Protein Atlas database (HPA, https://www.proteinatlas.org/) verified that protein levels of BST2 were also increased with increasing tumor grade (Uhlen et al., 2017). Immunohistochemical results indicated that BST2 protein is expressed in the cytoplasm and cell membrane, and immunofluorescence suggested that its subcellular localization is mainly in the Golgi apparatus and cell vesicles (De Palma and Lewis, 2013; Bego et al., 2015).

Considering that *BST2* expression was highest in the classical molecular subtype, we examined whether *BST2* high expression may be related to specific gene mutations (Figure 4G). Therefore, we downloaded the mutation data from the TCGA-GBM dataset. We divided cases on the basis of high and low expression of *BST2* and used the maftools package to identify potential associations between *BST2* and driver gene mutations (Figure 4H). In the *BST2* ^*high*^ tumors, the top 20 genes with the highest gene mutation frequency were *PTEN*, *AHNAK*, *KMT2C*, *APOB*, *SPTA1*, *PKHD1*, *HYDIN*, *ATRX*, *ADAM29*, *DNAH5*, *SYNE1*, *PIK3CA*, *NF1*, *RB1*, *RYR2*, *FLG*, *MUC16*, *TP53*, *EGFR*, and *TTN*. In the *BST2* ^*low*^ tumors, the top 20 genes with the highest gene mutation frequency were *TP53*, *PKHD1*, *HMCN1*, *TAF1L*, *RYR2*, *PIK3R1*, *APOB*, *IDH1*, *SYNE1*, *COL6A3*, *ATRX*, *FLG2*, *SPTA1*, *NF1*, *LRP2*, *MUC16*, *FLG*, *EGFR*, *TTN*, and *PTEN*. Among these gene mutations, *PTEN* and *TP53* showed the most significant differences between *BST2* ^*high*^ and *BST2* ^*low*^ tumors. In *BST2* ^*high*^ tumors, the *PTEN* mutation frequency was 31%. Mutation types in *PTEN* included the following: missense, nonsense, splice site, and frame shift deletions. However, the frequency of *PTEN* mutations in *BST2* ^*low*^ tumors was only 9%. In contrast, the mutation frequency of *TP53* was reversed relative to *PTEN* between the two expression groups: 8% in *BST2* ^*high*^ tumors and 36% in *BST2* ^*low*^ tumors.

### *BST2*-Related Genes Are Involved in Immune Response Related Pathways

**FIGURE 5:**
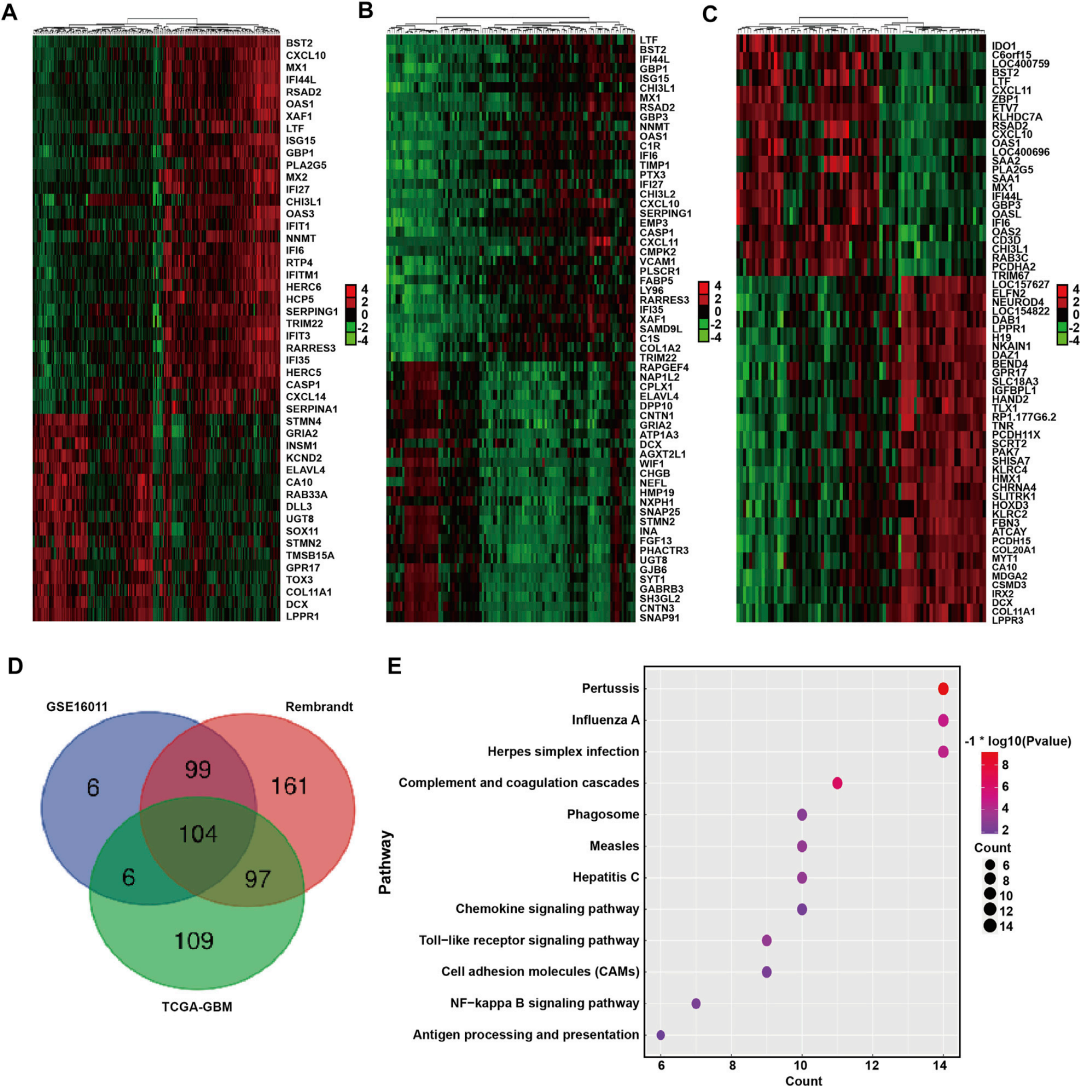
DEGs between BST2 high VS. low group related to KEGG immune pathways. Heatmaps generated using mRNA expression levels of partial DEGs based on *BST2* ^*high*^ and *BST2* ^*low*^ groups from **(A)** GSE16011, **(B)** Rembrandt and **(C)** TCGA-glioma datasets. Cutoff ratio: Log2 fold change is 1.5, 1.5, and 2.5, respectively; *p*-value < 0.05. **(D)** Venn diagram displaying the intersection of DEGs genes for the three datasets. **(E)** Bubble chart for KEGG pathway enrichment analysis of the intersecting genes.

We next identified and compared the DEGs associated with *BST2* expression in GBM patients in the GSE16011, Rembrandt-GBM and TCGA-GBM datasets (Log2 ∣fold change∣≥1, *p* < 0.05). The analysis yielded 215, 461, and 316 DEGs in GSE16011, Rembrandt-GBM and TCGA-GBM datasets, respectively (Figures 5A–C). Intersection of the DEGs from the three datasets yielded a total of 306 genes (Figure 5D). These resultant genes at the intersection of these three datasets were analyzed in David for KEGG pathway enrichment analysis to identify function (Figure 5E). We found that these genes were mainly enriched in immune response and inflammatory factor-related pathways, such as pertussis, influenza A, herpes simplex infection, toll-like receptor signaling, NF-kB signaling, antigen processing and presentation, chemokine signaling and complement and coagulation cascades.

### BST2 is Associated With Immune Cell Infiltration

**FIGURE 6:**
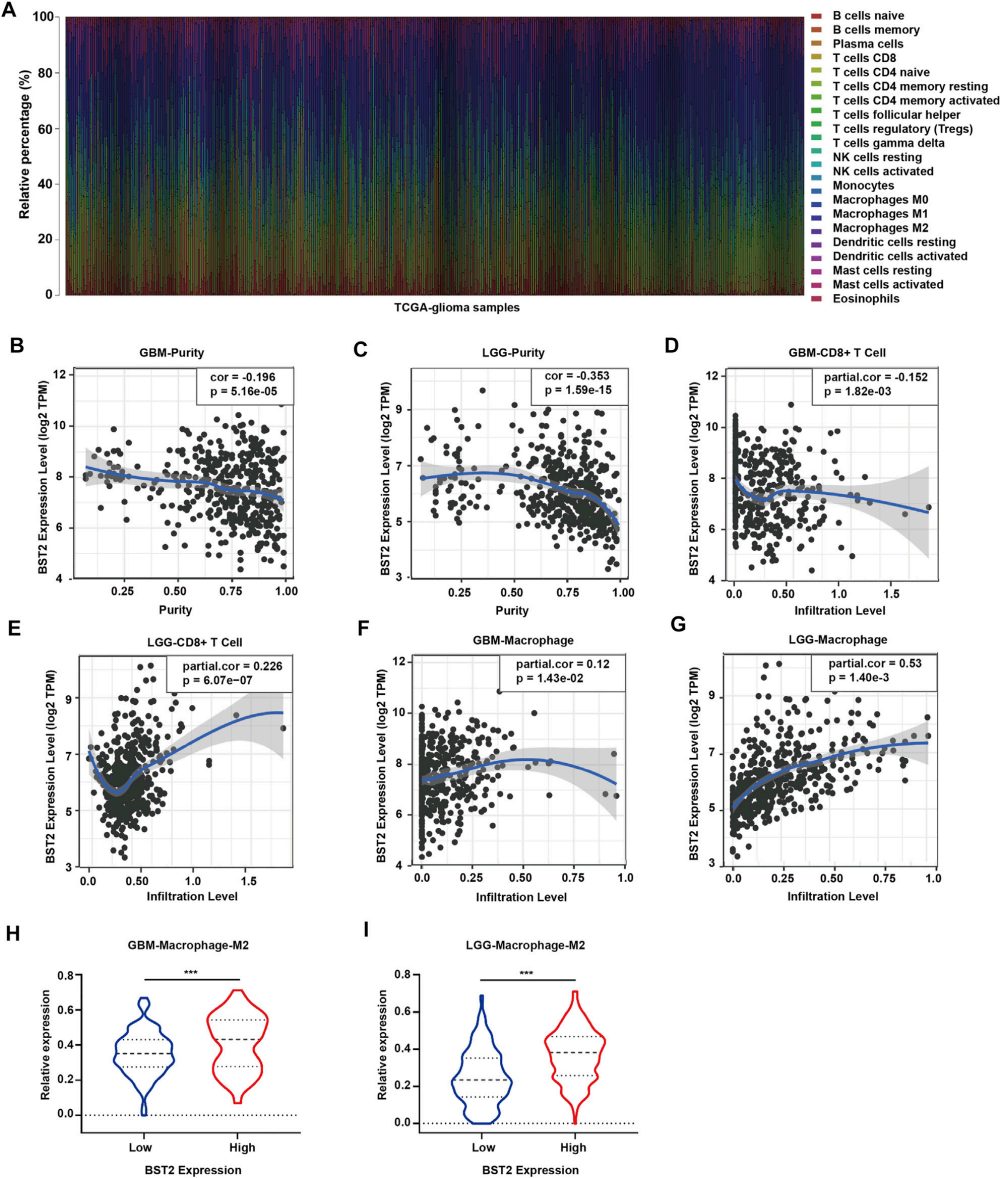
The mRNA expression level of *BST2* is associated with immune cells. **(A)** Stacked histogram reflects the relative expression of immune cells based on the mRNA expression profiles from the TCGA-glioma dataset. The correlation between the expression level of *BST2* and tumor purity is in **(B)** GBM and **(C)** LGG. The correlation between the expression level of *BST2* and the relative expression level of CD8^+^ T cells in GBM **(D)** and LGG **(E)**. The correlation between the expression level of *BST2* and the relative expression level of macrophages in GBM **(F)** and LGG **(G)**. The violin diagram reflects the expression value of M2 type macrophages in *BST2* ^*high*^ *and BST2* ^*low*^ GBM **(H)** and LGG **(I)** samples. ^∗∗∗^ *p* < 0.001.

Because *BST2* belongs to the MSigDB molecular tag and the enrichment analysis results revealed that it was related to immune response and cytokines, we explored the relationship of the gene with immune cell infiltration. We converted the expression profiles of the TCGA gliomas to the expression profiles of 22 immune cell types using CIBERSORT (Figure 6A). We also used TIMER to analyze the relationship between *BST2* expression and immune infiltration. We found that *BST2* was negatively correlated with tumor purity in GBM and low grade gliomas and (correlation coefficients −0.196 and −0.353) (Figures 6B,C). In GBM, the expression of *BST2* was negatively correlated with CD8^+^ T cells (correlation coefficient −0.152), while the expression of *BST2* in low grade glioma was positively correlated with CD8^+^ T cells (correlation coefficient 0.226) (Figures 6D,E). These results are consistent with the idea that a decrease in the number of CD8^+^ T cells may be related to the progression of gliomas and poor prognosis.

Macrophages were also positively correlated with GBM and low-grade gliomas (correlation coefficients were 0.12 and 0.53, respectively) (Figures 6F,G). Macrophages are critical players in the human innate immune system. However, under the influence of the tumor microenvironment, the gene expression of macrophages changes, yielding two types of cells, M1 type macrophages (classically activated macrophages, AMs) and M2 type macrophages (alternatively activated macrophages, AAMs) (Mantovani et al., 2002; Martinez et al., 2006). Based on the results of CIBERSORT, we found the M2 macrophage expression profile to be significantly increased in *BST2* ^*high*^ tumors for both GBM and low grade gliomas (Figures 6H,I).

### BST2 is Associated With Other Immune Checkpoint Molecules and Cytokine Genes

**FIGURE 7:**
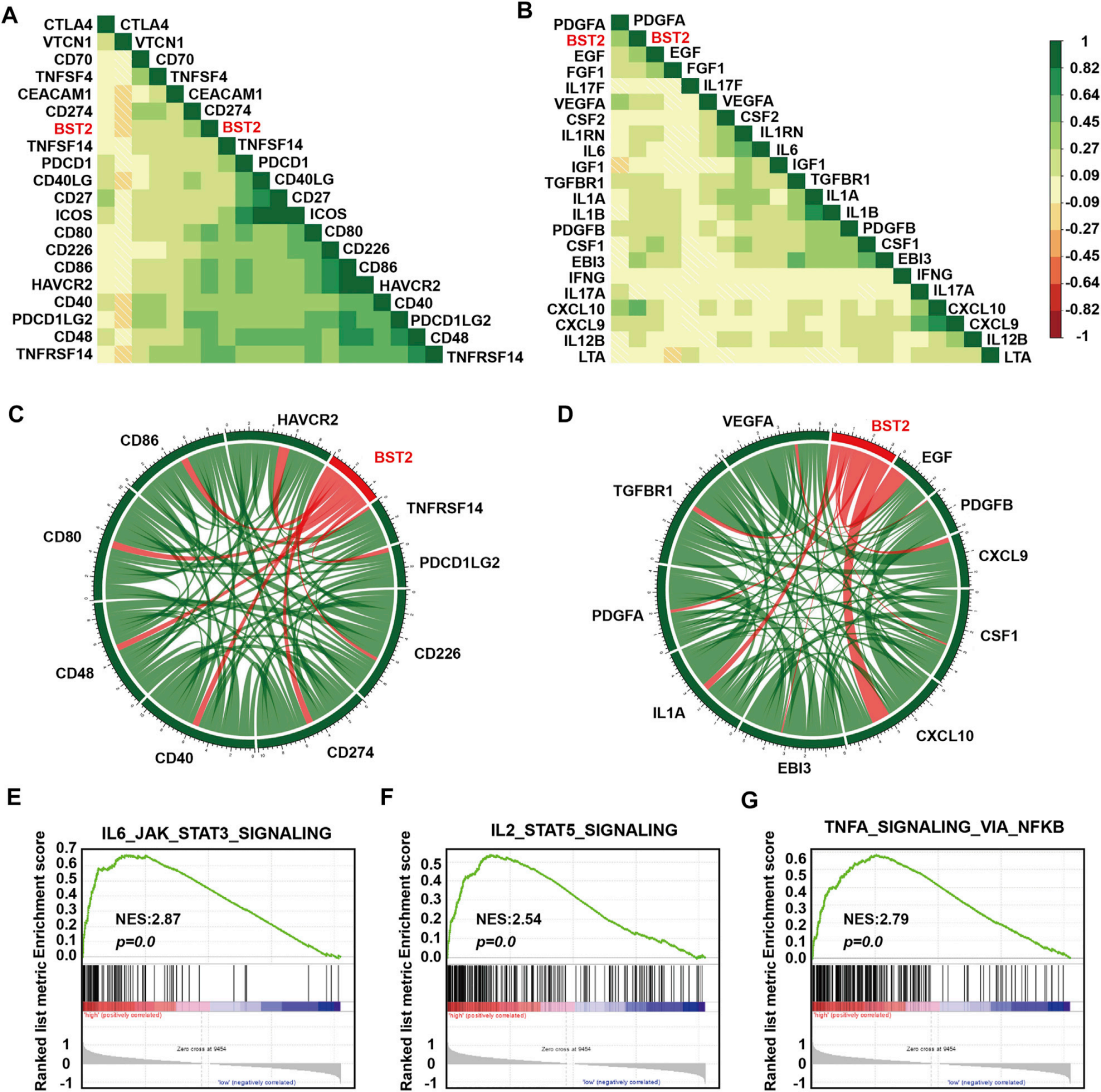
*BST2* is associated with immune checkpoint and cytokine genes. Corrgrams illustrating Pearson r-values for analysis between *BST2* and **(A)** immune checkpoint genes and **(B)** cytokine genes in the TCGA-glioma dataset. Chord diagram illustrating correlation between *BST2* and top **(C)** immune checkpoint genes and **(D)** cytokine genes in the TCGA-glioma dataset. **(E–G)**. GSEA enrichment analysis for IL6_JAK_STAT3_SIGNALING, IL2_STAT5_SIGNALING and the TNF-α signaling *via* NF-kB pathways pathway signatures in *BST2* ^*high*^ vs. *BST2* ^*low*^ samples in the TCGA GBM dataset. Normalized enrichment score (NES) and FDR are shown for each plot.

One of the characteristics of human tumor cells is that they avoid immune surveillance/destruction. One important mechanism is by affecting immune checkpoints that regulate the level of immune response activation on specific immune cells (Cantrell, 2002; Mantovani et al., 2002). In T cells, immune checkpoints can be divided into co-suppressive immune checkpoints and co-stimulatory immune checkpoints. The former checkpoints, such as PD1, CTLA-4 and VISTA, suppress immunity, and the latter checkpoints, such as CD28, ICOS, and CD137, promote immunity. Cancer cells cleverly influence immune checkpoints to evade immune surveillance. M2 type macrophages also participate in this suppression of the immune response process by secreting many cytokines, including IL-10 and TGF-β, which in addition to suppressing immune response, participate in tissue repair and matrix reconstruction, and promote tumor progression (Xu et al., 2003). We therefore calculated the correlation of the expression of *BST2* with immune checkpoint and cytokine genes in the TCGA-GBM data set (Figures 7A,B). Expression of *BST2* was closely related to the expression of the following immune checkpoint genes and chemokines: *CD226*, *CD274*, *CD48*, *CD40*, *CD80*, *CD86*, *HAVCR2*, *PDCD1LG2* and *TNFRSF14*; and *VEGFA*, *TGFBR1*, *PDGFA*, *IL1A*, *EBI3*, *CXCL10*, *CSF1*, *CXCL9*, *PDGFB* and *EGF* (Figures 7C,D). Finally, we used GSEA to predict that BST2 may promote GBM development through the IL6/JAK/STAT3 signaling, IL2/STAT5 signaling and TNF-α signaling *via* NF-kB pathways (Figures 7E–G).

### BST2 is Induced by IFN-γ

**FIGURE 8:**
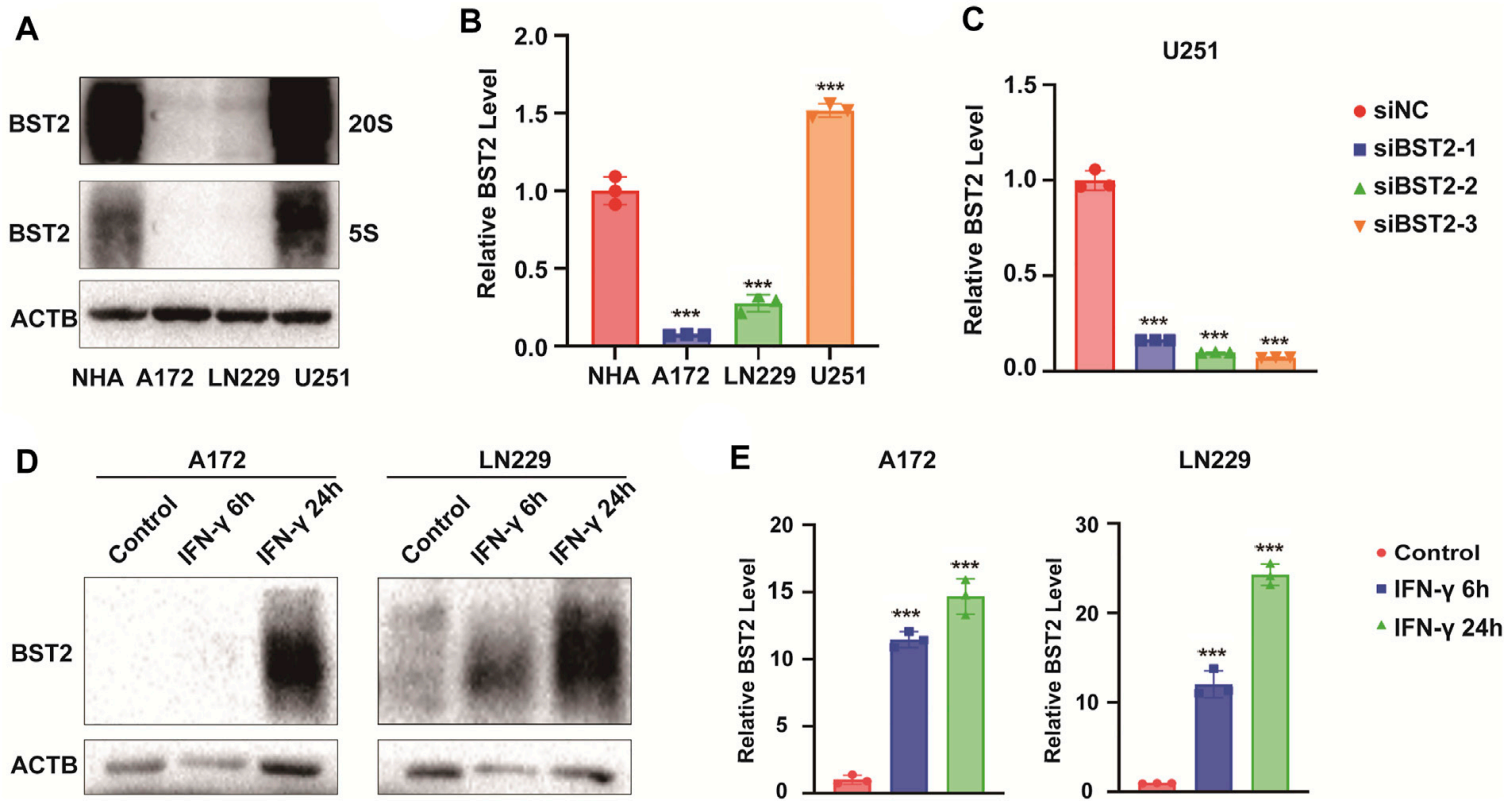
BST2 is induced by IFN-γ. **(A)**. Western blotting analysis of BST2 protein levels in NHA, A172, LN229 and U251 at two exposure durations. ACTB was used as an internal reference. **(B)** Relative quantitation histogram based on bands from western blots. **(C)** Graphic representation of qRT-PCR after siRNA knockdown of BST2. ACTB was used as an internal reference. **(D)** Western blotting analysis of BST2 protein levels in A172 and LN229 after IFN-γ treatment. **(E)** Relative quantitation histogram based on bands from western blots. ACTB was used as an internal reference. ^∗∗∗^ *p* < 0.001.

We first examined BST2 protein levels in NHA and GBM cell lines. Compared with NHA, BST2 protein expression was significantly higher in U251 but lower in A172 and LN229 cells lines (1.53 ± 0.07 vs. 1.01 ± 0.13, 0.10 ± 0.01 vs. 1.01 ± 0.13, 0.30 ± 0.08 vs. 1.01 ± 0.13, Figures 8A,B). U251 was chosen for knockdown experiments, whereas A172 and LN229 were used for IFN-γ induction experiments. qRCR analysis was used to verify the knockdown efficiency of BST2 (Figure 8C). After induction with IFN-γ (100 ng/ml), the protein expression of BST2 was significantly increased in a time-dependent manner in A172 and LN229 cells (×12 ×−23, Figures 8D,E).

### BST2 Promotes Proliferation of Glioblastoma Multiforme Cells *in vitro*

**FIGURE 9:**
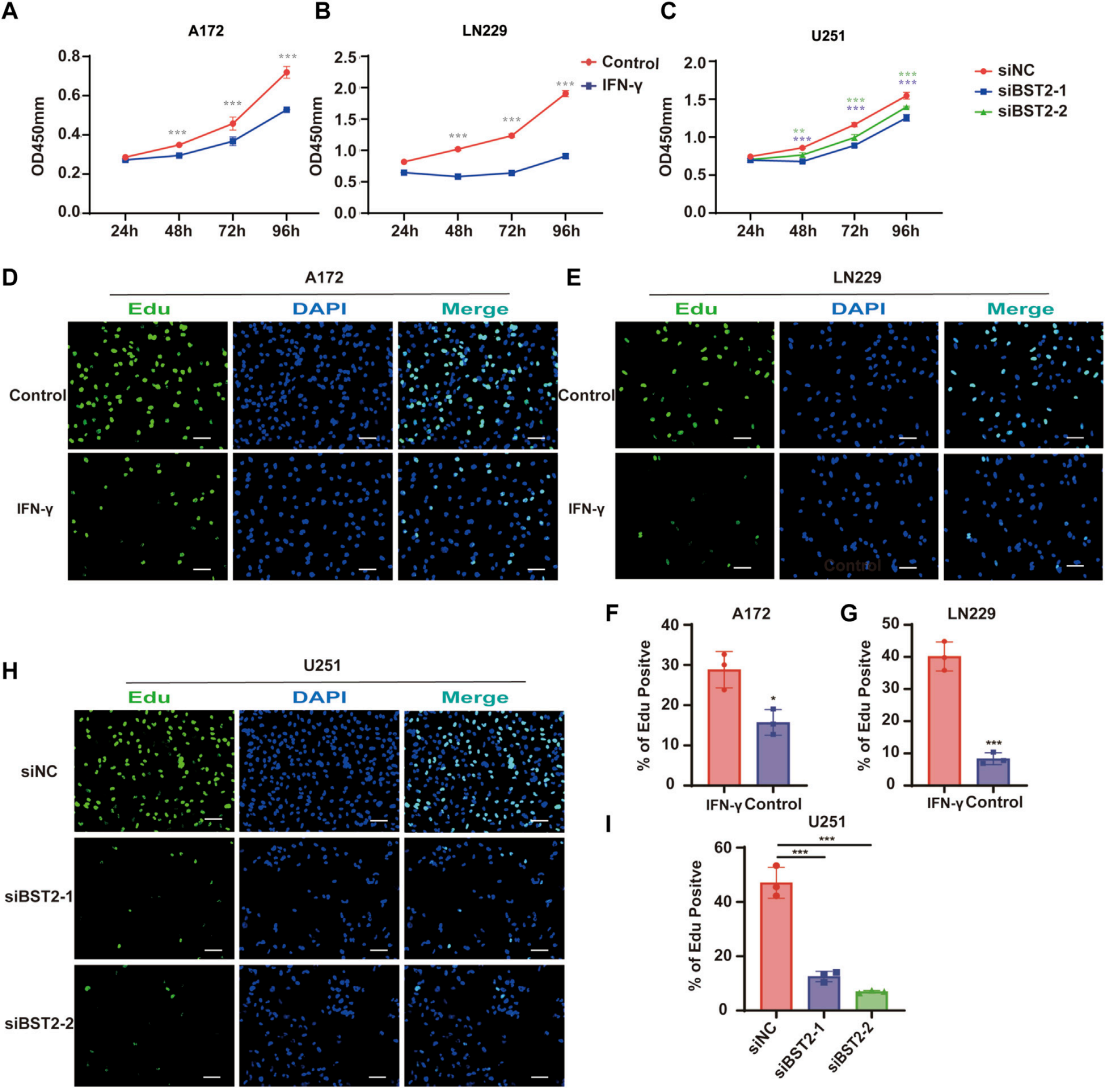
BST2 promotes proliferation of GBM cells *in vitro*. **(A,B)** Growth curves generated with data from the CCK8 assay after IFN-γ treatment in A172 and LN229 cell lines. **(C)** OD values from CCK8 assays of U251 plotted over time in hours after transfection of si-control and si-BST2. **(D,E)** Representative images of EdU assays after IFN-γ treatment of A172 and LN229 cell lines. **(F,G)** Graphic representation of EdU-positive cells in the above treatment groups. Scale bars: 20 µm. **(H)** Representative images of EdU assays after transfection of si-control and si-BST2 in U251 cells. Data are represented as the mean ± SD. **(I)** Graphic representation of EdU-positive cells in si-control and si-BST2 groups. Scale bars: 20 μm ^∗^ *p* < 0.05; ^∗∗∗^ *p* < 0.001.

To explore the effect of IFN-γ on GBM viability, GBM cells were treated with IFN-γ. Growth curves based on the CCK-8 assay demonstrated that IFN-γ significantly decreased cell growth in A172 and LN229 cells (Figures 9A,B). The results of the CCK8 assay demonstrated that the viability of U251 cells decreased relative to controls with BST2 knockdown (Figure 9C). The EdU assay confirmed these results. EdU incorporation in IFN-γ-treated A172 and LN229 cells decreased relative to controls (28.84 ± 4.52 vs. 15.69 ± 3.17 and 40.18 ± 4.51 vs. 8.36 ± 1.83, Figures 9D–G). Knockdown of BST2 led to a significant decrease in the percentage of EdU positive cells in U251 cells (Figures 9H,I).

### BST2 Promotes Glioblastoma Multiforme Cells Invasion

**FIGURE 10:**
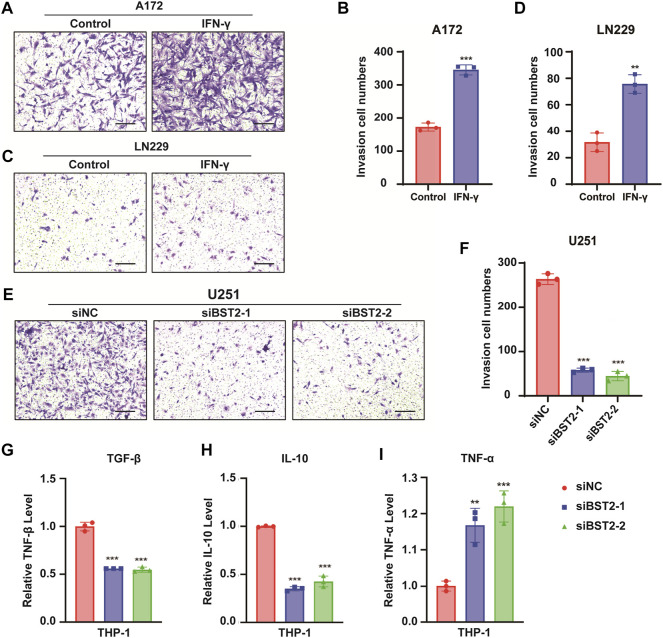
BST2 promotes GBM cells invasion. **(A,C)** Representative images of fixed and stained Transwell invasion assays performed on A172 and LN229 cells after treatment of IFN-γ. **(B,D)** Graphic representation of migrated and invaded cell counts from Transwell assay. Data are represented as the mean ± SD from three independent experiments. Scale bars:100 µm. **(E)** Representative images of Transwell invasion for U251 cells with siRNA knockdown of BST2. **(F)** Graphic representation of migrated cell counts from Transwell assays. Data are represented as the mean ± SD from three independent experiments. Scale bars:100 µm. **(G–I)** qRT-PCR for the expression levels of TGF-β, IL-10, and TNF-α in macrophages treated with preprocessed supernatant from U251 cells. ^∗∗^ *p* < 0.01; ^∗∗∗^ *p* < 0.001.

In Transwell invasion assays, treatment with IFN-γ led to an increased number of A172 and LN229 cells invading the membrane at 24 h (345.3 ± 15.04 vs. 172.7 ± 12.22, 75.67 ± 7.02 vs. 31.67 ± 7.02, Figures 10A–D). In contrast, the number of invaded cells was reduced by nearly 80% in U251 cells with BST2 knockdown (Figures 10E,F).

The supernatant of U251 cells in BST2 knockdown and control groups was collected and mixed 1:1 with complete culture medium to induce differentiation in THP-1 cells. THP1 cells were collected 48 h later, and macrophage program markers were detected by qRT-PCR. Results demonstrated that knockdown of BST2 decreased M2 polarization markers TGF-β and IL-10, but increased the M1 polarization marker TNF-α (Figures 10G–I).

## Discussion

Immunotherapy demonstrates the power of mining the tumor microenvironment for new molecular targets in the treatment of human cancer. However, human gliomas reside in a unique tumor microenvironment, attracting both intrinsic and peripheral immune cell types. The molecular characterization of GBMs with an emphasis on their tumor microenvironment may therefore yield approaches to immunotherapy with greater efficacy in the treatment of human gliomas. Here, we distinguished glioma tumor samples on the basis of tumor purity using expression data to identify DEGs and the biological pathways they are associated with. Our analysis yielded several genes involved in IFN signaling, such as *BST2*. High expression of *BST2* was associated with low tumor purity, increasing tumor grade and decreased numbers of CD8^+^ T cells in GBM. We found the gene to be associated with other immune checkpoint genes, which highlights BST2 as another molecular target for immunotherapy tailored for human gliomas.

Numerous studies show that interferons inhibit tumor growth. IFN-γ has been shown to effectively inhibit the growth of tumor cells by inducing G1 phase arrest, apoptosis (Höpfner et al., 2004), and metastasis (Sun et al., 2021). Interferons also have a role in tumor immunosuppression. IFN-γ promoted the accumulation of CXCR3^+^ lymphocytes in tumor tissues, which exhibited anti-tumor immune properties (Li et al., 2018). IFN-γ also directly up-regulated functional MHC class I molecules on human GBMs, which led to increased immunogenicity (Yang et al., 2004). In contrast, suppression of the production of IFN and other proinflammatory cytokines through BST2-ILT7 interactions in plasmacytoid dendritic cells may contribute to tumor tolerance (Cao et al., 2009). Therefore, IFN-γ treatment of GBM and the role of BST2 in IFN-γ treatment warrant further study.

The IFN-inducible factor BST2 has emerged as an important component of the antiviral immune response. BST2 is a negative regulator of the type I IFN signaling pathway and inhibits the release of diverse enveloped viral particles from infected cells (Perez-Caballero et al., 2009; Swiecki et al., 2012). Many factors have been implicated in the up-regulation of BST2 expression, notably IFN-α (Jones et al., 2013), IFN-γ (Yoo et al., 2011), and even viral infections (Homann et al., 2011). However, chronic infection with Mouse mammary tumor virus has also been shown to suppress both IFNα and IFN-γ, while BST2 remains elevated in breast cancer (Jones et al., 2013). BST2 expression is also significantly higher in various tumors than in the corresponding normal tissues (Wainwright et al., 2011; Sayeed et al., 2013; Kuang et al., 2017).

BST2 function has been previously investigated in a variety of human cancers. BST2 has been shown to activate EGFR and the initiation of downstream signaling pathways by regulating its association with lipid rafts in hepatocellular carcinoma and to be involved in resistance to tumor cell death and chemotherapy (Zhang et al., 2019b). BST2 has also been proposed as a tumor-associated antigen in some tumor cell lines (Gong et al., 2015; Yang et al., 2018). Finally, the expression of BST2 was found to be increased during brain tumor progression in an orthotopic mouse tumor model where it was mainly located in the cell membrane and cytoplasm (Wainwright et al., 2011). All together, these results indicate that interfering with BST2 may be a valuable strategy in the treatment of GBM. In fact in B-cell lymphoma, targeting BST2 with two independent monoclonal antibodies delayed tumor growth in a syngeneic mouse model of the disease (Gong et al., 2015). Other studies have also demonstrated that inhibiting BST2 can achieve the effect of inhibiting tumor cell growth or inducing cell death (Sayeed et al., 2013; Mahauad-Fernandez and Okeoma, 2017). However, BST2 silencing and immunotherapy did not improve the overall survival of tumor bearing mice in an orthotopic mouse brain tumor model (Wainwright et al., 2011). This result might be possibly due to the fact that GBM is protected from normal immune surveillance because of the blood-brain-barrier, the lack of lymphatic drainage and low expression of MHC II expression. Therefore, the role of BST2 in the development of GBM requires further study.

Immune checkpoint inhibitors have achieved remarkable outcomes in the treatment of a variety of solid human tumors. Checkpoint inhibitors target the immunomodulatory effect of cytotoxic T lymphocyte–associated protein 4 (CTLA-4) and programmed death-1 (PD-1)/programmed death-ligand 1 (PD-L1) restoring effector T-cell function and antitumor activity (Smyth et al., 2016; Ramos-Casals et al., 2020). CTLA-4 is mainly expressed on activated T cells, which binds to CD80 and transmits inhibitory signals to cause immunosuppression. Tumor-associated macrophages/microglia (TAMs) are a major stromal cell component in GBM(37). PD-1 is expressed by human TAMs and inhibits tumor immunity. We found that *BST2* was positively correlated with several immune checkpoint genes, including *PD1*, *ICOS*, *CD80*, *CD226*, *CD86*, *HAVCR2*, *CD40*, *PDCD1LG2*, *CD48*, and *TNFRSF14*. The expression of *BST2* was positively correlated with the expression of M2 type macrophages in all grades of gliomas, but the amount of CD8^+^ T cells exhibited inconsistent results, which highlights the complexity of the microenvironment of gliomas. A series of clinical trials investigating efficacy of checkpoint inhibitors in GBM showed that only a small subset of patients (8%) demonstrated objective responses (Buerki et al., 2018). One possibility for this result is the lower tumor mutational burden of GBM (Brennan et al., 2013) and the low level of T-cell infiltration (Woroniecka et al., 2018). Since IFN induces the immunogenicity of GBM and promotes the infiltration of immune cells, treatment with IFN might improve the efficacy of immune checkpoint inhibitors in GBM patients. Finally, considering the relationship between *BST2* and these immune checkpoint genes, the inhibition of BST2 may also improve the efficacy of immune checkpoint inhibitors in the treatment of GBM.

Other genes related to IFN-γ may also promote the development of human GBM. Ly6E is a member of a multigene family of glycosylphosphatidylinositol-anchored cell surface proteins and is considered a stem/progenitor cell marker (Bacquin et al., 2017). Overexpression of Ly6E promoted cancer cell growth and metastasis in gastric cancer (Lv et al., 2018), drug resistance and immune escape in breast cancer, and TGF-β-induced PD-L1 activation and binding of NK cells to cancer cells (AlHossiny et al., 2016). An LY6E antibody drug conjugate (ADC) was found to inhibit cell proliferation and produced long-lasting tumor regression in multiple solid tumor xenograft models (Asundi et al., 2015). LGALS3BP has been found to be elevated in the serum of patients with cancer. The protein might have a role in centriole biogenesis, NK cell immune response and cell-matrix interactions. Expression of the 90K immunostimulatory antigen LGALS3BP has been inversely correlated with the tumorigenicity of mammary carcinoma, GBM, and other tumor-derived cell lines in athymic mice (Fogeron et al., 2013; Stampolidis et al., 2015; Zhou et al., 2015). Thus, several of these correlated genes may be important in the study of human GBM.

In summary, we analyzed the GBM expression data in the TCGA, Rembrandt and GSE16011 datasets and found tumor purity to be closely related to prognosis. *IFN* was one of the differentially expressed genes between high and low tumor purity in GBM. A gene correlated with IFN expression was *BST2* which we found to be positively correlated with multiple immune checkpoint genes and negatively correlated with tumor purity, CD8^+^ T cells, and M2 type macrophages. Finally, a series of experiments confirmed that the expression of BST2 can be significantly induced by IFN, and knockdown of BST2 can significantly inhibit the growth and invasion of GBM cells. Loss of BST2 also affects the phenotype of tumor-associated macrophages. Thus, targeting BST2 might be considered as a viable therapeutic strategy in the treatment of human GBM.

## Data Availability

The original contributions presented in the study are included in the article/supplementary material, further inquiries can be directed to the corresponding author.
